# Characterization of humoral and cell-mediated immunity in rabbits orally infected with *Encephalitozoon cuniculi*

**DOI:** 10.1186/s13567-020-00806-9

**Published:** 2020-06-15

**Authors:** Edita Jeklova, Lenka Leva, Jan Matiasovic, Petra Ondrackova, Vladimir Kummer, Martin Faldyna

**Affiliations:** Veterinary Research Institute, Hudcova 296/70, 621 00 Brno, Czech Republic

## Abstract

Encephalitozoonosis is a common infectious disease widely spread among rabbits. *Encephalitozoon cuniculi*, is considered as a zoonotic and emerging pathogen capable of infecting both immunocompetent and immunocompromised hosts. The aim of the study was to describe in detail the spread of the *E. cuniculi* in a rabbit organism after experimental infection and the host humoral and cellular immune response including cytokine production. For that purpose, healthy immunocompetent rabbits were infected orally in order to simulate the natural route of infection and euthanised at 2, 4, 6 and 8-weeks post-infection. Dissemination of *E. cuniculi* in the body of the rabbit was more rapid than previously reported. As early as 2 weeks post-infection, *E. cuniculi* was detected using immunohistochemistry not only in the intestine, mesenteric lymph nodes, spleen, liver, kidneys, lungs and heart, but also in nervous tissues, especially in medulla oblongata, cerebellum, and leptomeninges. Based on flow cytometry, no conspicuous changes in lymphocyte subpopulations were detected in the examined lymphoid organs of infected rabbits. Cell-mediated immunity was characterized by ability of both CD4^+^ and CD8^+^ T cells to proliferate after stimulation with specific antigens. Th1 polarization of immune response with a predominance of IFN-γ expression was detected in spleen, mesenteric lymph nodes and Peyer’s patches. The increased expression of IL-4 and IL-10 mRNA in mixed samples from the small intestine is indicative of balanced control of IFN-γ, which prevents tissue damage. On the other hand, it can enable *E. cuniculi* to survive and persist in the host organism in a balanced host-parasite relationship. The Th17 immunity lineage seems to play only a minor role in *E. cuniculi* infection in rabbits.

## Introduction

Encephalitozoonosis is a common cause of morbidity and mortality in pet and conventionally raised rabbits. Its causative agent, *Encephalitozoon cuniculi*, is a member of the phylum Microspora, which includes ubiquitous, eukaryotic, single-celled, spore-forming, obligate intracellular parasites [1]. Once considered as protozoa, genome sequence analysis has supported reclassification of microsporidia as fungi [2]. Despite wide host distribution among mammals, including rodents, carnivores, birds and primates, *E. cuniculi* primarily affect rabbits. This pathogen is also considered as a zoonotic opportunistic pathogen in immunocompromised people [3, 4].

In rabbits, horizontal transmission by ingestion or inhalation of spores occurs most frequently [1], but intrauterine [5, 6] and ocular infections have also been documented [7–9]. After ingestion, *E. cuniculi* invades the intestinal epithelium by extruding the polar filament. Sporoplasm is transferred through this polar filament directly into the host cells, where parasites multiply by merogony and by sporogony. Infective spores or proliferative forms are disseminated throughout the body via infected macrophages or by release into the blood [10]. Organs with high blood flow such as kidneys, lungs and liver are the first target for *E. cuniculi* in rabbits. Nevertheless, the final predilection sites are kidneys and the brain [11]. From 35 days after infection, spores are excreted intermittently in the urine of infected rabbits for up to 3 months if not longer [7, 11].

Even in an immunocompetent host, *E. cuniculi* persists despite an active immune response. However, latent infection remains asymptomatic as long as the parasite multiplication and the host immune response are balanced [12]. In latent infections, gross lesions are rare or even absent except in cases of chronic interstitial nephritis. *E. cuniculi* infection is associated with focal, granulomatous lesions mainly in the brain and kidneys. If immune competence is compromised, microsporidia can proliferate rapidly and clinical disease may occur [7, 13]. Rabbits suffering from encephalitozoonosis may clinically display neurological symptoms, signs of kidney failure or phacoclastic uveitis. In many cases, the onset of clinical signs is sudden and often follows a stressful situation [14, 15].

Data obtained from murine models have shown that cell-mediated immunity is critical for protection against *E. cuniculi* infection. Whereas CD8^+^ T cells are essential for protection against an intraperitoneal infection, both CD4^+^ and CD8^+^ T lymphocyte subpopulations play a substantive protective role in the oral route of infection entry [16]. IFN-γ is a crucial cytokine for the survival of mice infected through either the intraperitoneal or oral route, apparently for its ability to polarize the development of adaptive immunity towards a Th1 response, promoting the generation of CD8^+^ T cell immunity [17]. Minimal Th2 cytokine production has been observed during the infection of mice with *E. cuniculi* [18]. Elevated serum IFN-γ levels have recently been detected in naturally infected rabbits [19].

Microsporidial infection induces specific antibody production, and persistence of antibodies in serum reflects latent infection. The infectivity of microsporidia is reduced by treatment with immune serum and complement in vitro and specific antibodies contribute to resistance to *E. cuniculi* infection by facilitating spore opsonization, complement fixation and ingestion of opsonized spores by macrophages [20, 21]. Nevertheless, the humoral immune response does not appear to be protective, as immune serum does not prevent lethal disease in athymic mice [12].

Despite the fact that encephalitozoonosis in laboratory and pet rabbits is of clinical significance worldwide [4], studies of the immune response to this infection are based mainly on a murine model. The aim of this study was to characterize the spread of the pathogen and the cellular immune response in immunocompetent rabbits after experimental oral infection with *E. cuniculi*.

## Materials and methods

### Preparation of *E. cuniculi* spores

Spores of a rabbit strain of *E. cuniculi* (CH-K-2169; kindly provided by Prof. P. Deplazes, University of Zurich, Switzerland) were produced on the RK 13 cell line in minimal essential medium with antibiotics (10 U/mL penicillin; 0.1 mg/mL streptomycin and 0.25 μg/mL amphotericin) and 5% fetal bovine serum. The spores were harvested, resuspended in the culture medium, and stored at 4 °C. Spores were purified by density gradient centrifugation in Percoll (Sigma-Aldrich, St. Louis, MO, USA) using a standard procedure [22]. The viability of *E. cuniculi* was verified by inoculation with subsequent multiplication of an infection dose aliquot on the RK 13 tissue culture.

### Animals and experimental design

Outbred New Zealand White SPF European rabbits, strain Crl:KBL (free of common rabbit pathogens including *E. cuniculi*) were obtained from the Charles River Laboratories Germany. The rabbits were housed individually in wire-mesh cages in the animal care facility under controlled conditions at the Veterinary Research Institute, Brno, Czech Republic. Housing conditions were described elsewhere [7]. The animals were housed and handled with the agreement of the Institutional Commission for Animal Welfare. The experiment was performed in compliance with the Act No. 246/1992 Coll. of the Czech National Council on the protection of animals against cruelty, and with the agreement of the Branch Commission for Animal Welfare of the Ministry of Agriculture of the Czech Republic.

A total of 25 four-month-old male rabbits were sedated (0.03 mg/kg medetomidine and 3 mg/kg ketamine) and then 20 rabbits were infected, using a stomach tube, with 4 × 10^7^*E. cuniculi* spores in 1 mL of PBS and 5 control rabbits were administered only PBS. Uninfected control rabbits were housed in a separate room. After infection, the rabbits were clinically monitored on a daily basis (assessment of behaviour, posture, movement) for physical activity and for any clinical signs of disease. Based on the results of a previous experiment [7], five infected and one control rabbits were anaesthetized (0.1 mg/kg medetomidine and 15 mg/kg ketamine) and samples of non-heparinized and heparinized peripheral blood form the central ear artery and cerebrospinal fluid (CSF) from the cerebellomedullary cistern were obtained at 2, 4, 6 and 8 weeks post-infection (pi). Rabbits were euthanised subsequently and tissue samples were collected immediately.

### Histopathology and immunohistochemistry

In order to determine the prevalence of tissue lesions and the spread of *E. cuniculi* throughout the host body, the following tissue samples were fixed in 10% neutral buffered formalin: spleen, mesenteric lymph node, liver, kidneys, stomach, small intestine, appendix, lungs, heart, testis, cerebrum, cerebellum, medulla oblongata, lumbar medulla and leptomeninges. For histopathology, paraffin sections (6 µm) were stained with haematoxylin and eosin. Extensive histopathological changes throughout the tissue were defined as severe; focal changes as moderate; and occasional areas of increased cellularity, or perivascular or periportal cuffing were determined as mild.

For immunohistochemistry, serial sections were mounted on silanized slides, deparaffinized in xylene (3 × 5 min), hydrated in a series of graded ethanol, and washed in Tris–HCL buffer (pH 7.6). Subsequently, heat-induced antigen retrieval was performed in a microwave on high power (750 W) for 15 min (3 × 5 min) in citrate buffer, pH 6.0. Endogenous peroxidase activity was blocked using 3% H_2_O_2_ for 15 min. The mouse monoclonal antibody (EC11C5; dilution 1:100; [23]) was used for the detection of *E. cuniculi*. The sections were incubated overnight at 4 °C. EnVisionTM^+^/HRP, Mouse (DakoCytomation, Glostrup, Denmark) and 0.03% DAB (3,3′diaminobenzidine, Sigma-Aldrich, St. Louis, MO, USA) as a chromogen (10 min at room temperature) were used for visualization of the immunoreaction complexes. The slices were counterstained with haematoxylin. Stained sections were dehydrated and mounted under glass coverslips in Entellan (Merck, Darmstadt, Germany). Staining was performed in parallel with positive and negative controls.

### Detection of spores in urine and CSF

Urine samples were collected during necropsy by urinary bladder puncture. Calcium carbonate was removed from urine by 99% acetic acid [24]. In order to release DNA, the spore wall was destroyed by mechanical disruption (MagNA Lyser Instrument, Roche, Basel, Switzerland) with zirconia silica beads (0.1 mm) and total DNA from 0.2 mL of treated urine or 0.2 mL of CSF was isolated using DNeasy 96 Blood and Tissue kit (Qiagen, Valencia, CA, USA). PCR was performed with the LightCycler LC480 (Roche, Basel, Switzerland). The whole procedure was described in detail elsewhere [7].

### Serological testing

In order to define humoral response, specific anti-*E. cuniculi* IgM and IgG in serum of infected rabbits at respective pi intervals were detected using ELISA as described previously [25]. For preparation of a soluble antigen, spores were mixed with solid glass beads (≤ 106 μm; Sigma-Aldrich, St. Louis, MO, USA) and sonicated on ice (30 min, 60 W). For characterization of antibody response, absorbances (iEMS Reader, Labsystems, Helsinki, Finland) read at dilutions 1:30 for IgM and 1:100 for IgG in serum samples were used.

### Cell isolation

For the determination of cell immune response, the spleen, mesenteric lymph nodes, Peyer’s patches and popliteal lymph nodes were collected into RPMI 1640 medium (Sigma-Aldrich, St. Louis, MO, USA). To define the total number of cells in each organ, the weight of the tissues was determined at the beginning of the cell isolation process. Cell suspensions were prepared by careful teasing the lymphoid tissue using two forceps. All cell suspensions were filtered through a fine nylon mesh and erythrocytes contaminating lymphoid organ suspensions were lysed with a haemolytic solution (8.26 g NH_4_Cl, 1 g KHCO_3_ and 0.037 g EDTA per 1L of distilled water). The numbers of the isolated cells were expressed as the cells per gram of tissue.

### Immunostaining and flow cytometry analysis

For flow cytometry, the isolated cells were washed in a washing and staining buffer (WSB-PBS with 0.2% gelatine from cold water fish skin, 0.1% sodium azide and 0.05 mM EDTA, all reagents from Sigma-Aldrich, St. Louis, MO, USA), resuspended in WSB and adjusted to a density of 5 × 10^6^/mL. In samples of peripheral blood, erythrocytes were lysed by a haemolytic solution before adding of primary antibodies. A panel of antibodies used for immunostaining are listed in Table 1. All antibodies were previously titrated to define optimal working dilutions. Furthermore, isotype-matched controls were included for each labelling. For surface markers staining, 50 μL cell suspensions were incubated with 50 μL of unconjugated primary antibodies and 20 μL of inactivated goat serum at room temperature for 15 min. After washing in WSB, mixtures of goat anti-mouse conjugates of appropriate subisotypes (Table 1) were used as the secondary antibodies and after another 20 min incubation at 4 °C, the cells were washed and resuspended in WSB. For 3-colour labelling, cells were then incubated with 200 μL of 10% inactivated mouse serum for 10 min at 4 °C and subsequently, after washing, a directly conjugated antibody was added for another 20 min at 4 °C. In both types of surface staining, 20 μL of propidium iodide were added for exclusion of nonviable cells for the last 5 min of incubation. After another washing, the cells were resuspended in WSB and analysed.

**Table 1:** **Antibody panel used for immunostaining of peripheral blood and lymphoid organ cell suspensions for flow cytometry analysis in a rabbit after oral infection with*Encephalitozoon cuniculi.***

No. of tube	CD molecule	Isotype	Clone	Source	Secondary antibody
1	pT	IgG1	RTH21A	VMRD Inc., USA	R-PE
	CD4	IgG2a	MCA799G	Serotec	AF 647
	CD8-FITC	IgG1	MCA1576F	Serotec	–
2	IgM	IgG1	MCA812G	Serotec	R-PE
3	CD14	IgG1	CAM36A	VMRD Inc., USA	FITC
	CD45	IgG2a	ISC18A	VMRD Inc., USA	R-PE
4	CD79α-PE	IgG1	HM57	DakoCytomation, DK	–

Goat anti-mouse conjugates of appropriate subisotypes (Southern Biotechnology Assoc. Inc., Birmingham, USA) were used as secondary antibodies. Appropriate isotype-matched controls were added to separate tubes for each antibody used.

For CD79α intracellular staining, cell suspensions were fixed and permeabilized using IntraStain kit (DakoCytomation, DK) and labelled according to a protocol recommended by the producer.

Data was acquired on a flow cytometer BD FACS Aria Fusion operated with BD FACSDiva software (BD Biosciences, Franklin Lakes, NJ, USA). Gating was based on forward angle and right angle scatter signals. The common leukocyte antigen CD45 and CD14 expression was used for the lymphogate setup and lymphocyte purity determination as described previously [26]. Percentage values of lymphocyte subsets were also recalculated to absolute cell numbers per 1 g of tissue or 10^6^ per 1 mL of peripheral blood cells.

### Proliferation assay

Detection of proliferation activity of whole blood and lymphoid organ cell suspensions was based on incorporation of ^3^H-thymidine as described previously [7]. For the lymphocyte proliferation assay, the density of the cell suspension was adjusted to 1 × 10^6^/mL in RPMI 1640 medium supplemented with 10% precolostral calf serum, 100 000 U/L penicillin and 0.2 g/L streptomycin. After 5 days of in vitro lymphocyte stimulation (37 °C, 5% CO_2_) with live *E. cuniculi* spores (4 × 10^5^/well) or non-specific mitogens phytohaemagglutinin (PHA, 100 μg/mL), concanavalin A (ConA, 10 μg/mL) and pokeweed mitogen (PWM, all mitogens purchased from Sigma-Aldrich, St. Louis, MO, USA, 10 μg/mL) or without stimulant, 50 μL of medium with ^3^H-thymidine (5 μCi/mL) was added for the last 20 h. The incorporation of ^3^H-thymidine was analyzed with a microplate scintillation and luminescence counter (TopCount NXT™, Packard Bioscience Company, Meriden, CT, USA). The results were expressed in terms of stimulation indices (SI), which were calculated as the ratio of counts per minute (CPM) in stimulated samples versus CPM in non-stimulated ones.

### CFSE labelling

For tracking of antigen-specific lymphocyte proliferation in the spleen, a fluorescent dye, carboxyfluorescein succinimidyl ester (CFSE) was used. For this purpose, 1 × 10^7^ splenocytes were suspended in 1 mL DPBS (Lonza Walkersville, Walkersville, MD, USA) prior to addition of 1 mL of a 10 μM CFSE-solution (Invitrogen, Carlsbad, CA, USA) in DPBS. After vortexing and incubation for 7 min at room temperature, CFSE staining was stopped by adding 2 mL FCS and pelleting by centrifugation at 1500 RPMI for 10 min. Excessive CFSE was removed by two additional washing steps in culture medium. CFSE-labelled splenocytes (2 × 10^5^/well) were either cultivated in cell culture medium or specifically stimulated with *E. cuniculi* spores (4 × 10^5^/well). After 6 days of incubation (37 °C, 5% CO_2_), splenocytes were harvested and stained with specific anti-rabbit monoclonal antibodies for multicolour flow cytometry analysis (Table 2). For determination of the absolute cell number, the BD TruCOUNT Tubes (BD Biosciences, Franklin Lakes, NJ, USA) were used in this experiment. The absolute numbers of proliferating CD4^+^, CD8^+^, panT^+^ and IgM^+^ lymphocytes for each rabbit were determined by detection of CFSE intensity for parental, early proliferating and late proliferating cells and the absolute values of antigen-stimulated splenocytes of a respective lymphocyte subpopulation were divided by the value of the same cell subpopulation from the control medium in respective stages in order to obtain a stimulation index (SI).

**Table 2:** **Antibody panel used for immunostaining of CFSE-labelled splenocytes after specific stimulation with*E. cuniculi*spores or cultivation in cell culture medium only.**

No. of tube	CD molecule	Isotype	Clone	Source	Secondary antibody
1	CD8	IgG2a	IS27A	VMRD Inc., USA	R-PE
	CD4	IgG1	RTH1A	VMRD Inc., USA	AF 647
2	pT	IgG1	RTH21A	VMRD Inc., USA	R-PE
3	IgM	IgG1	MCA812G	Serotec	AF 647
4	CD14	IgG1	CAM36A	VMRD Inc., USA	AF 647
	CD45	IgG2a	ISC18A	VMRD Inc., USA	R-PE

Goat anti-mouse conjugates of appropriate subisotypes (Southern Biotechnology Assoc. Inc., Birmingham, USA) were used as secondary antibodies. CFSE-labelled cells without any primary or secondary antibodies and with appropriate isotype-matched controls were added to separate tubes for each antibody used and included in flow cytometry analysis.

### Detection of cytokines

For the detection of IFN-γ, IL-4, IL-10 and IL-17 cytokines, samples of the spleen, mesenteric lymph nodes, popliteal lymph nodes, Peyer’s patches and mixed samples of the duodenum, jejunum and ileum (hereinafter referred to as small intestine) were collected into RNAlater Solution (Invitrogen, Carlsbad, CA, USA) and stored at − 20 °C until used. Tissue samples were homogenised (MagNA Lyser Instrument, Roche, Basel, Switzerland) with zirconia silica beads (2.3 mm) in TRI Reagent-RT (MRC, Cincinnati, OH, USA) and RNA was isolated according to the manufacturer’s instructions. Total RNA was reverse transcribed with M-MLV reverse transcriptase (200U) (Invitrogen, Carlsbad, CA, USA) using oligo-dT primers (Generi Biotech, Hradec Kralove, Czech Republic) and stored at − 20 °C until used.

Real-time PCR was performed with the LightCycler 480 (Roche, Basel, Switzerland). Primers for cytokines and reference genes were designed using NCBI primer designing tool Primer–blast and synthesized by Generi Biotech (Hradec Kralove, Czech Republic). The sequences of used primers were: IFN-γ: 5′-GGA TGA CTT CCA AAA TCT GAC TCG-3′ and 5′-TTC ACT TAC TGC TTT ACG CTG GAC-3′; IL-4: 5′-TTC TAC CTC CAC CAC AAG GTG TC-3′ and 5′-GAG TCC TCT CAG GAG TCT GAG GTC-3′; IL10: 5′-TTC TGT GCC TGA CCA CAC TTT C-3′ and 5′-CTA GGA GTC TCT GGA ACA CTC GG-3′; IL17: 5′-ACC ACA TGA ACT CTG TCC CAA TC-3′ and 5′-CCT ACA GCC ACC AGC ATC TTC-3′. Three candidate reference genes (HPRT, GAPDH and HMBSe) were tested using GeNorm [27] and the HPRT was selected as the most stable gene in our experiment (HPRT primers: 5′-TGA AAC TGG AAA AGC AAA ATA CAA AG-3′ and 5′-CGA TGT CAA TGA GAC TCC TGA TG-3′). Each reaction was carried out in a total volume of 10 µL, which consisted of QuantiTect SYBR Green PCR Kit (Qiagen, Valencia, CA, USA), 0.1 µM of each relevant primer and 1 µL of template DNA. Cycling conditions were as follows: 15 min of initial denaturation at 95 °C was followed by 40 cycles consisting of 95 °C for 20 s, 58 °C for 30 s and 72 °C for 30 s. Each run included a non-template control to test the assay reagents for contamination. Analysis of the melting temperature of the PCR products was performed on all samples. The relative expression of a gene of interest was calculated as a ratio to HPRT reference gene using the following formula: [1/(2CtGOI)])/[1/(2CtHPRT)]. In order to determine the prevailing Th immune response, the ratios of Th1 cytokine (IFN-γ) versus Th2 (IL-4 and IL-10) and Th-17 (IL-17) cytokines were calculated.

### Statistical analysis

Because of relatively small numbers of experimental animals in respective groups, Mann–Whitney non-parametric test was used for comparisons of results from infected animals of respective groups with control non-infected rabbits. Differences with the value of *P* < 0.05 were considered statistically significant. All calculations were performed with GraphPad Prism^®^ software version 3.03 (GraphPad Software, La Jolla, CA, USA).

## Results

### Clinical signs

Clinical examination of all infected rabbits did not reveal any pathology, including changes in the behaviour, throughout the experimental period.

### Histopathology and immunohistochemistry

During the infection, mild, moderate and occasionally severe microscopic lesions compatible with encephalitozoonosis were seen mainly in the lungs, liver, kidneys, spleen and central nervous tissue of infected rabbits. No histopathological lesions were found in control rabbits. Histological examination of the lungs from infected rabbits revealed mild to severe alveolar inflammatory infiltrations of lymphocytes, plasma cells and monocytes and alveolar destruction. Liver lesions consisted of mild to moderate periportal and perivascular infiltrations by lymphocytes and plasma cells. Kidney lesions were characterized by mild to severe tubular degeneration and interstitial infiltrates of mononuclear cells with lymphocytes. In the spleen, hyperaemia of the red pulp and lymphocyte infiltration were observed. Lesions in nervous tissue included mild to moderate tissue and perivascular lymphocyte infiltrates. No lesions that could be directly attributed to *E. cuniculi* were seen in the intestine of any rabbit. Severity of histological lesions during the infection are presented in Table 3.

**Table 3:** **Severity of histological lesions in the lungs, liver, kidneys, spleen and central nervous tissue (CNS) during the respective weeks post-infection (pi) in rabbits (from 5 animals in respective weeks) experimentally orally infected with*Encephalitozoon cuniculi.***

Week pi	Lungs	Liver	Kidneys	Spleen	CNS
2	+++	+	+	++	+
4	++	++	++	++	++
6	+	++	+++	++	+
8	+	++	++	+	+

Extensive histopathological changes throughout the tissue were defined as severe (+++); focal changes as moderate (++); and occasional areas of increased cellularity, or perivascular or periportal cuffing were determined as mild (+).

**Figure 1:**
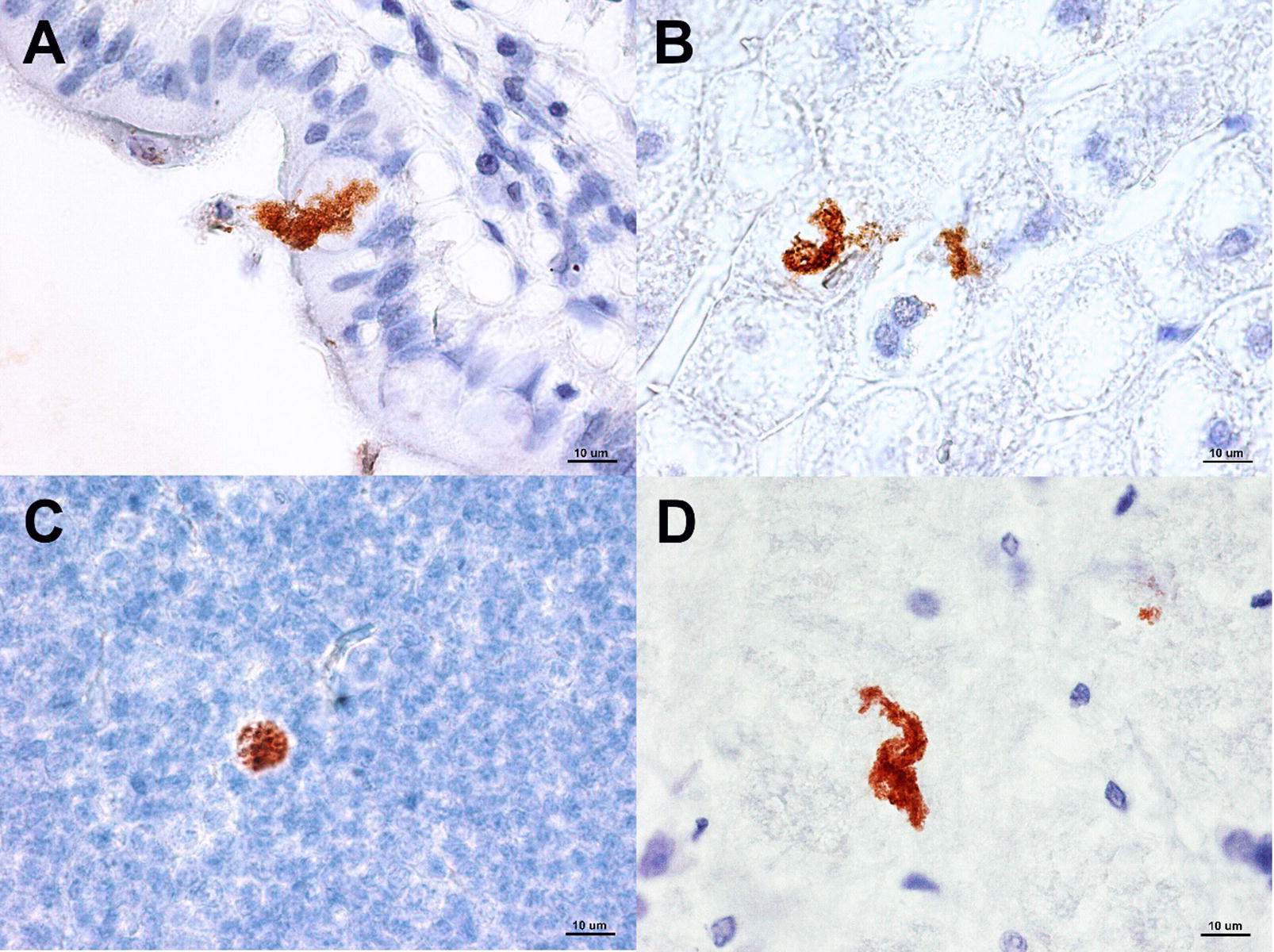
**Identification of*E. cuniculi*using immunohistochemistry (with specific monoclonal anti—*E. cuniculi*antibody EC11C5) in various organs in experimentally orally infected rabbits. A** small intestine (ileum); **B** liver; **C** mesenteric lymph node; **D** cerebrum.

Using immunohistochemistry, the presence of proliferative stages or spores of *E. cuniculi* was detected in all the examined tissue samples. Examples of positive detection of the *E. cuniculi* organism in various organ tissues are presented in Figure 1. As early as 2 weeks pi, *E. cuniculi* was detected not only in the intestine, mesenteric lymph nodes, spleen, liver, kidneys, lungs and heart, but also in nervous tissue including the cerebrum, cerebellum, medulla oblongata, lumbar medulla and leptomeninges. Nevertheless, the number of rabbits with positive detection of spores in various nervous tissue using this method increased in later terms pi. Nervous tissues with the most frequently detected microsporidia were cerebrum, medulla oblongata and leptomeninges. Numbers of rabbits with positive detection of the *E. cuniculi* in various tissues in respective weeks pi are shown in Table 4. In control uninfected rabbits, no *E. cuniculi* organism was detected.

**Table 4:** **Results of immunohistochemistry examination using monoclonal antibody EC11C5 in rabbits experimentally orally infected with*Encephalitozoon cuniculi.***

Week pi	Stomach	Small intestine	Appendix	Spleen	Mesenteric lymph node	Liver	Lung	Heart
2	2	4	3	2	5	4	4	2
4	4	5	3	4	4	4	5	3
6	2	5	4	4	4	4	4	3
8	0	5	4	2	4	5	4	2

Week pi	Kidney	Cerebrum	Cerebellum	Medulla oblongata	Lumbar medulla	Leptomeninges	Testis
2	4	1	2	3	1	2	2
4	5	4	3	4	3	3	3
6	4	4	1	4	3	2	2
8	3	4	3	5	2	4	4

Numbers of infected rabbits from 5 animals in respective weeks with positive detection of *E. cuniculi* in various tissues in respective weeks post-infection (pi).

### Detection of spores in the urine and CSF

Using PCR method, *E. cuniculi* spores were for the first time detected in a sample of urine from one rabbit as soon as at 4 weeks pi, then in one sample from a rabbit at 6 weeks pi and in two samples obtained from rabbits at 8 weeks pi. All samples of CSF were negative for the presence of the *E. cuniculi* DNA.

### Humoral immune response

In all respective terms pi, strong IgM and IgG antibody responses were detected in the sera of infected rabbits. Significant increases (*P* < 0.01) in absorbance values of IgM and IgG antibodies when compared with the response of non-infected rabbits were recorded in all terms pi.

### Detection of lymphocyte subpopulations

No conspicuous changes in lymphocyte subpopulations were detected in the examined lymphoid organs of infected rabbits when compared with control uninfected animals. In peripheral blood, a significant increase in absolute numbers of CD8^+^ lymphocytes (*P* < 0.05) was noted 6 weeks pi. A significant decrease in absolute numbers of pT^+^ cells (*P* < 0.05) was detected in the spleen 2 and 4 weeks pi. In the mesenteric lymph nodes, a significant increase in absolute numbers of CD4^+^8^+^ lymphocytes (*P* < 0.05) at 6 weeks pi were ascertained. No changes were detected in absolute numbers of lymphocyte subpopulations in Peyer’s patches and in popliteal lymph nodes.

### Proliferation assay

Lymphocyte proliferation as a response to specific stimulation with *E. cuniculi* spores was determined. When SI of infected and uninfected rabbits were compared, a significant increase in proliferation activity was revealed in whole blood samples 4 weeks pi (*P* < 0.05), in splenocytes 4 (*P* < 0.05), 6 (*P* < 0.01) and 8 (*P* < 0.01) weeks pi and in cells isolated from popliteal lymph nodes 6 weeks pi (*P* < 0.01). No elevation of specific proliferation activity was detected in lymphocytes from mesenteric lymph nodes and Peyer’s patches. In order to determine non-specific proliferation activity, cells were also stimulated with plant mitogens. A significant decrease in proliferation ability was recorded 4 weeks pi in splenocytes stimulated with PWM (*P* < 0.01) and in Peyer’s patch lymphocytes stimulated with PHA (*P* < 0.05) and PWM (*P* < 0.05).

### Phenotyping of proliferative cells

**Figure 2:**
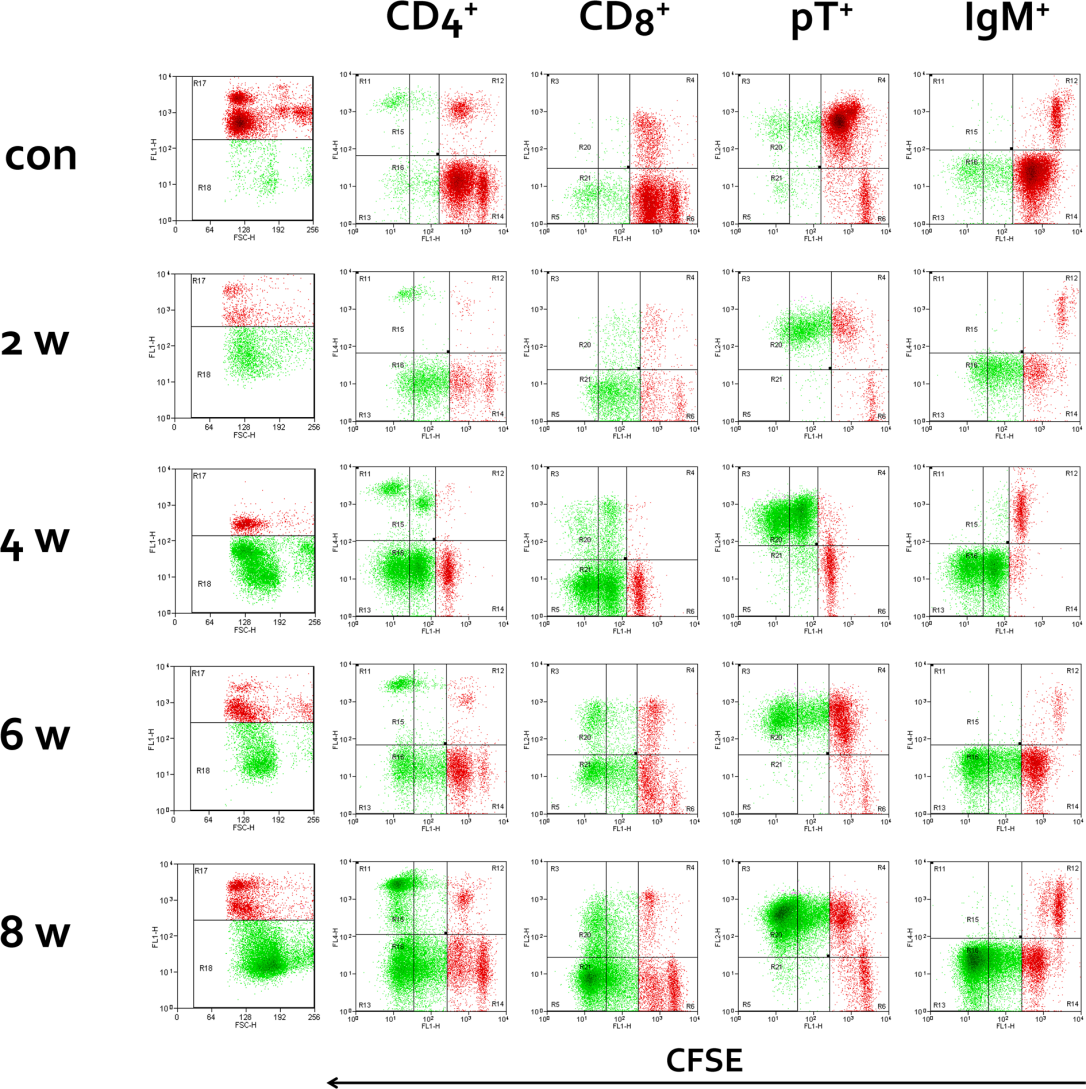
**Representative flow cytometry analysis of CFSE labelled splenocytes in control uninfected rabbit (con) and rabbits in 2, 4, 6 and 8** **weeks after*E. cuniculi*oral infection.** Splenocytes were stimulated with *E. cuniculi* spores and after 6 days of incubation stained with specific monoclonal antibodies against cell surface markers. Relative numbers of parental (red) and early and late proliferating cells (green) for each lymphocyte subpopulation were determined in all animals and then, using the BD TruCount Tubes (BD Biosciences, Franklin Lakes, NJ, USA), recalculated to absolute numbers in order to obtained stimulation indices (SI).

In order to determine the phenotype of lymphocytes responsible for specific proliferation, splenocytes were labelled using CFSE and stimulated with *E. cuniculi* spores, with subsequent staining of surface lymphocyte markers. Flow cytometric staining profiles of splenocytes from infected and uninfected rabbits in respective weeks pi are shown in Figure 2. In all respective post-infection terms, both populations of T lymphocytes (CD4^+^ and CD8^+^) significant strongly responded to antigen stimulation with their early and late proliferation in *E. cuniculi* infected rabbits (Figure 2). Only low values of SIs were detected in uninfected rabbits. When SIs in respective lymphocyte subpopulations at 2 weeks pi were compared, proliferation of CD4^+^ cells was noticeably higher than proliferation of CD8^+^ splenocytes. At 4 weeks pi, proliferation of CD4^+^ and CD8^+^ cells was comparable and at 6 and 8 weeks pi, proliferation of CD8^+^ T cells exceeded proliferation of CD4^+^ lymphocytes. Significantly higher early and late proliferation of IgM^+^ cells was detected only in splenocytes at 8 weeks pi (Table 5).

**Table 5:** **Comparison of stimulation indices (SI) in parental and early and late proliferated CD4+; CD8+; pT+ and IgM+ carboxyfluorescein succinimidyl ester (CFSE) labelled splenocytes in control uninfected rabbits (con) and rabbits 2, 4, 6, 8** **weeks after*E. cuniculi*oral infection.**

	CD4+ cells (SI)			CD8+ cells (SI)			pT+ cells (SI)			IgM+ cells (SI)		
Week pi	Parental population	Proliferating		Parental population	Proliferating		Parental Population	Proliferating		Parental Population	Proliferating	
		Early	Late		Early	Late		Early	Late		Early	Late
con	1.1	1.2	1.8	1.0	1.3	1.2	0.8	1.0	1.7	1.0	1.3	1.6
2	21.9	1351.6*	7450.2*	2.7	65.9*	59.3*	2.0	66.5*	105.6*	3.1	16.6*	6.1
4	1.4	10.1	92.2**	2.8*	7.6*	107.1**	2.2	5.1*	64.3**	1.5	4.7	7.0
6	0.7	7.8*	29.8*	1.2	8.3*	96.3*	0.6	5.5**	33.3**	1.4	3.4	2.3
8	1.2	36.3**	712.4**	2.1	24.2**	1016.7**	0.9	23.2**	695.2**	3.0*	44.0**	15.3**

Data are presented as mean of SI from 5 animals in each group. For tracking of cell proliferation, CFSE labelled cells were incubated with *E. cuniculi* spores for 6 days and then stained with monoclonal antibodies against rabbit lymphocytes. The absolute numbers of respective subpopulations of lymphocytes for each rabbit were determined by detection of CFSE intensity for parental, early proliferating and late proliferating cells and the absolute values of antigen-stimulated splenocytes of respective lymphocyte subpopulations were divided by the value of the same cell subpopulation from the control medium in respective stages in order to obtain a SI. The parameters with significant difference (*P* < 0.05) and (*P* < 0.01) when compare to control (con) uninfected rabbits are marked with * and ** respectively.

### Cytokine production

**Figure 3:**
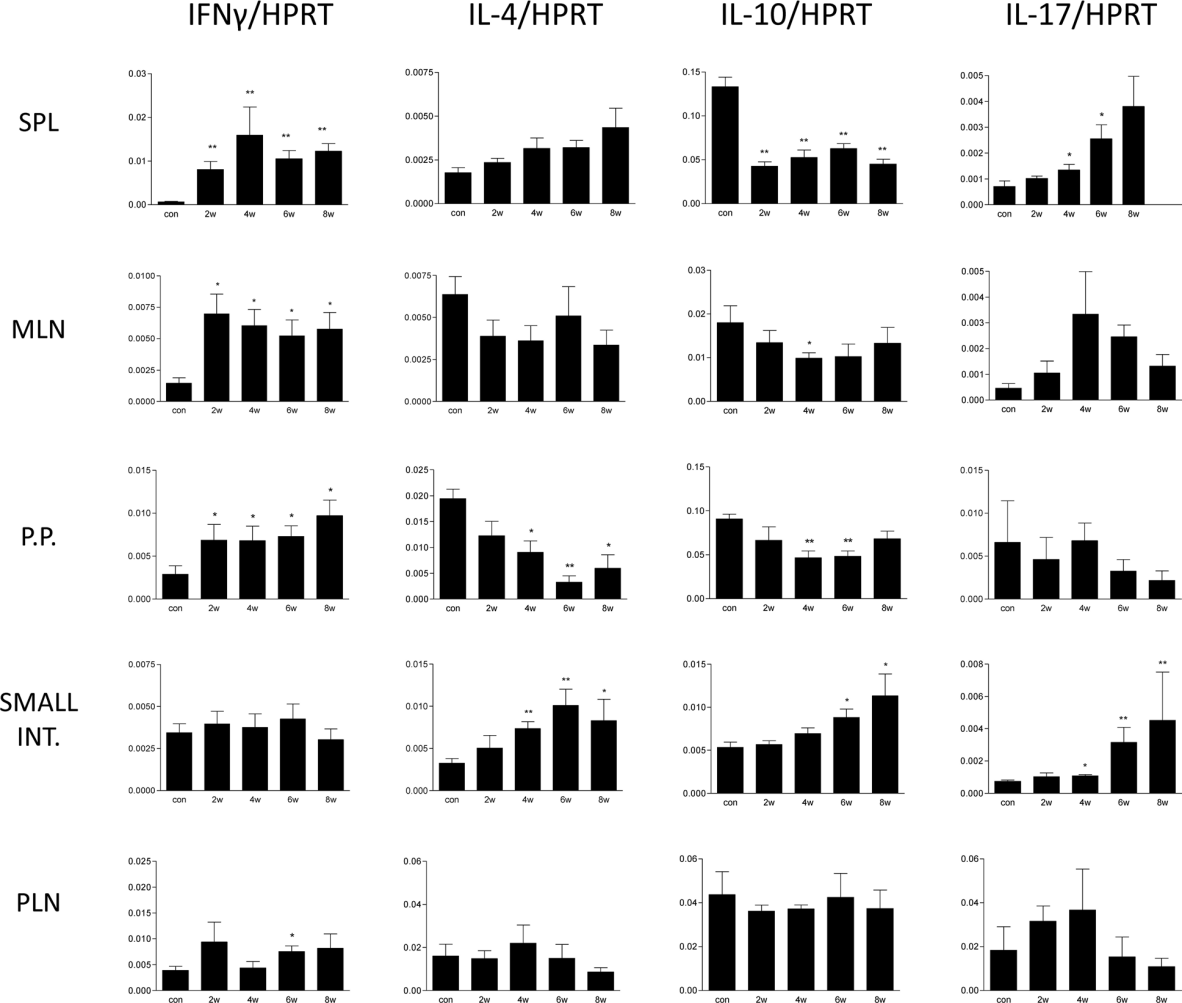
**Cytokine production in various organs of*E. cuniculi*experimentally orally infected rabbits in 2, 4, 6 and 8** **weeks (w) post-infection.** Real-time PCR was performed in samples of spleen (SPL), mesenteric lymph nodes (MLN), Peyer’s patches (PP), small intestine and popliteal lymph node (PLN). The relative expression of a gene of interest (IFN-γ, IL-4, IL-10 and IL-17) was calculated as a ratio to HPRT reference gene. The parameters with significant difference (*P* < 0.05) and (*P* < 0.01) when compare to control (con) uninfected rabbits are marked with * and ** respectively. Data are presented as mean ± SD from 5 animals in respective week post-infection.

All tested cytokine mRNAs were found at detectable levels. When compared with uninfected rabbits, levels of mRNA for IFN-γ in the spleen were significantly elevated (*P* < 0.01) and levels of IL-10 were significantly reduced (*P* < 0.01) at all times pi (Figure 3). The levels of IL-17 were increased at 4 and 6 (*P* < 0.05) weeks pi. Significant changes in mRNA for IL-4 were not detected in this compartment. In the mesenteric lymph nodes, increased production of IFN-γ at all times pi (*P* < 0.05) was also recorded. The levels of IL-10 tended to decrease and were significantly lower (*P* < 0.05) at 4 weeks pi. No changes in mRNA for IL-4 and IL-17 were detected. Significantly elevated levels of IFN-γ at all times pi (*P* < 0.05) were ascertained also in Peyer’s patches. The levels of mRNA for IL-10 were significantly reduced (*P* < 0.01) at 4 and 6 weeks pi and IL-4 mRNA at 4 (*P* < 0.05), 6 (*P* < 0.01) and 8 (*P* < 0.05) weeks pi. In contrast to that, when compared to uninfected animals the levels of IFN-γ were not changed in the small intestine. Moreover, levels of IL-10 mRNA were elevated (*P* < 0.05) at 6 and 8 weeks pi and levels of IL-4 and IL-17 were elevated at 4 (*P* < 0.01, *P* < 0.05), 6 (*P* < 0.01) and 8 (*P* < 0.05, *P* < 0.01) weeks pi. In the popliteal lymph nodes, only a significantly increased level of IFN-γ at 6 weeks pi was detected.

**Figure 4:**
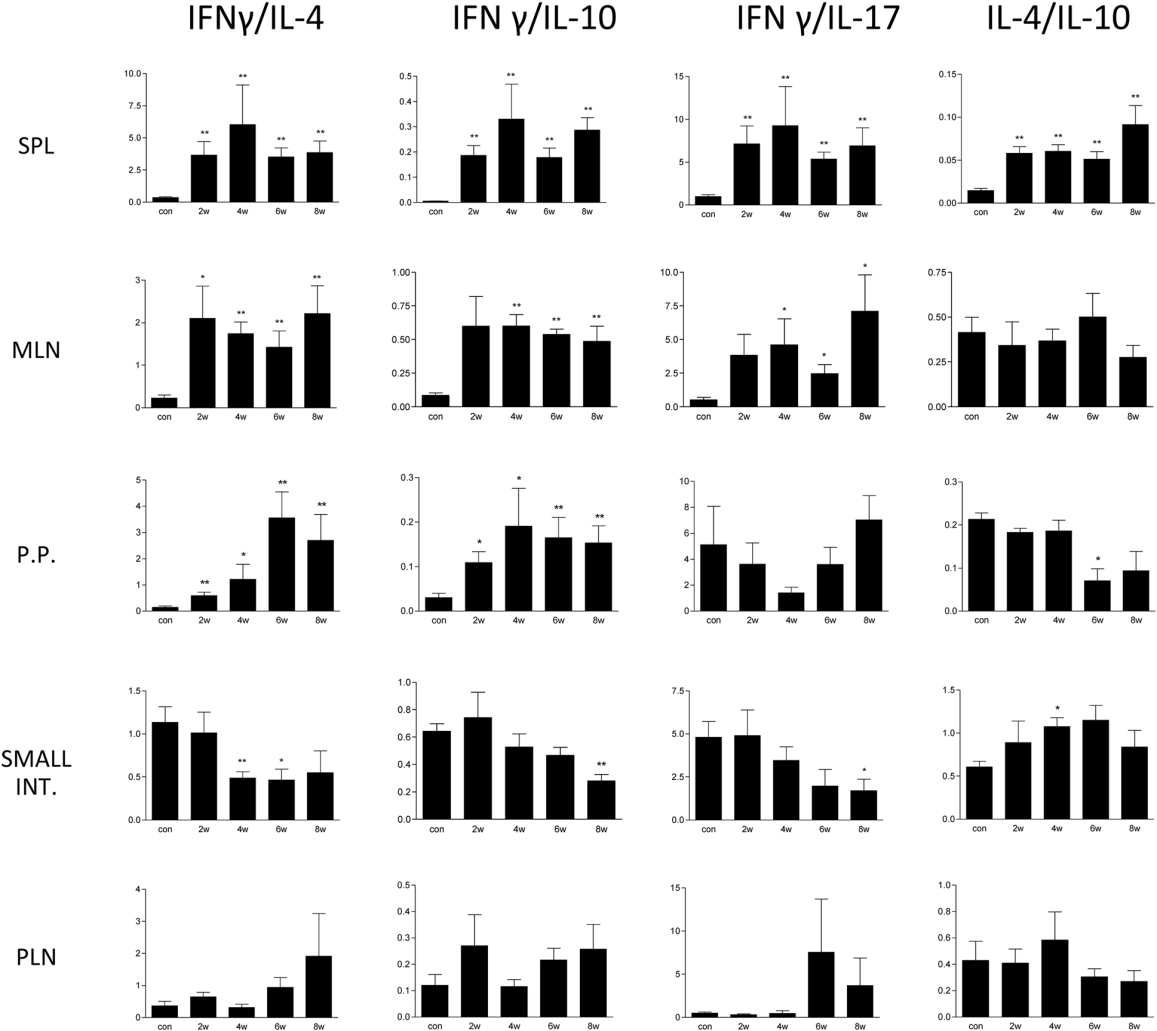
**Prevailing Th immune response in various organs of*E. cuniculi*experimentally orally infected rabbits in 2, 4, 6 and 8** **weeks (w) post-infection.** Real-time PCR was performed in samples of spleen (SPL), mesenteric lymph nodes (MLN), Peyer’s patches (PP), small intestine and popliteal lymph node (PLN). The ratios of Th1 cytokine (IFN-γ) versus Th2 (IL-4 and IL-10) and Th-17 (IL-17) cytokines were calculated. The parameters with significant difference (*P* < 0.05) and (*P* < 0.01) when compare to control (con) uninfected rabbits are marked with * and ** respectively. Data are presented as mean ± SD from 5 animals in respective week post-infection.

Based on the calculation of the cytokine ratio, Th1 immune response significantly prevailed (*P* < 0.01) over Th2 and Th17 in the spleen for the entire experiment (Figure 4). Similar results were obtained in mesenteric lymph nodes and Peyer’s patches. In contrast to that, in the small intestine, Th2 cytokine IL-4 predominated at 4 (*P* < 0.01) and 6 (*P* < 0.05) weeks pi and IL-10 (*P* < 0.01) together with IL-17 (*P* < 0.05) at 8 weeks pi.

## Discussion

Encephalitozoonosis is a common infectious disease widely spread among rabbits. Due to its clinical importance in the rabbit population and the considered zoonotic potential of *E. cuniculi* [4], a detailed study of this microsporidial infection in rabbits is of scientific interest. Nevertheless, most studies examining infection course and immune response to *E. cuniculi* infection have utilized murine models [28]. The present study described in detail the spread of the *E. cuniculi* in a rabbit organism after experimental oral infection and the host humoral and cellular immune response, including cytokine production.

Severity of clinical signs is dependent on the immunocompetence of the host and virulence of the microsporidia. In the present study, healthy immunocompetent rabbits were infected orally in order to simulate the natural route of infection. In agreement with previous studies [7, 29], the course of experimental encephalitozoonosis in immunocompetent rabbits was subclinical and no neurological, renal or ocular signs of disease were observed during the entire experimental period. However, as described previously, subclinical latent encephalitozoonosis can be reactivated and become clinically manifested after a short-term immunosuppression [7].

In contrast to rabbits with clinical symptoms, in immunocompetent chronically infected rabbits, severe histopathological lesions are rare or absent and the pathogen may occur even in isolated cells devoid of any inflammatory reaction [13]. Histopathological lesions noticed in present study were usually of moderate or mild severity that correspond to previous studies in immunocompetent hosts [7, 30]. Using polyclonal immune serum and direct immunofluorescence, Cox et al. [11] reported that at 31 days after experimental infection, the kidneys, lungs and liver were the major target organs for *E. cuniculi* in rabbits, whereas the heart was affected to a lesser extent and the brain was totally unaffected. At 98 days pi, *E. cuniculi* organisms were frequently observed in the brain and kidneys and, in one case, in the heart. At that time, lesions and the parasite were disappearing from other organs. In present study, the dissemination of *E. cuniculi* throughout the host body was detected using immunohistochemistry with a specific monoclonal antibody. As early as 2 weeks pi, *E. cuniculi* was detected not only in the intestine, mesenteric lymph nodes, spleen, liver, kidneys, lungs and heart, but also in at least one case in the brain and other nervous tissues. During the course of infection, the pathogen persisted in all examined organs except stomach. However, the occurrence of microsporidia in nervous tissue increased. Nervous tissues with the most frequently detected *E. cuniculi* were cerebrum, medulla oblongata and leptomeninges. Similarly, in pet rabbits with suspected encephalitozoonosis due to exacerbation of clinical signs, the most affected brain regions were cerebrum and leptomeninges [31].

Excretion of spores in urine can be considered as the primary mode of dissemination for *E. cuniculi*. Spores excreted in urine can usually be detected at 5 [7] or 6 weeks pi [11]. Since the excretion of the spores is intermittent, a negative result does not exclude infection. In the present study, the spores in urine were detected in one rabbit as early as 4 weeks pi.

Over the entire post-infection period, all samples of CSF were negative for *E. cuniculi* DNA. Similar results were obtained in rabbits at 18 weeks after experimental oral and ocular *E. cuniculi* infection by Jeklova et al. [7] and by Künzel at al. [32] who tested CSF in 12 pet rabbits with suspected encephalitozoonosis. On the other hand, Jass et al. [24] obtained positive results in two of 19 CSF samples from pet rabbits with clinical encephalitozoonosis. Nevertheless, we can assume that detecting the presence of spores in CSF is not a useful tool for intravital diagnosis of encephalitozoonosis.

As in other intracellular infections, immune responses to microsporidia are both cellular and humoral. Although humoral response alone is not considered to be protective, the antibodies contribute to host resistance [12, 33]. On the other hand, serological testing remains the most important tool for *intra vitam* diagnosis of encephalitozoonosis in rabbits. Simultaneous testing of IgM and IgG specific antibodies gives an indication of the infection status. The presence of IgM antibodies is indicative of active infection. The presence of only IgG specific antibodies denotes chronic/latent infection [25]. In the present study, specific IgM and IgG antibodies were detected in all infected rabbits 2 weeks pi, and both antibody isotypes persisted up to 8 weeks.

The cell-mediated immune response plays a principal role in the prevention of lethal encephalitozoonosis [18]. The important role of T-cell immunity in *E. cuniculi* infection has been described based on experimental infections in a murine model. While CD80+ T lymphocytes are essential for protection after intraperitoneal infection, both CD4+ and CD8+ T lymphocyte subpopulations play a substantive protective role in the case of a peroral, i.e. natural route of infection [16]. In mice, T cell immune response to oral infection was reflected by an elevation of the absolute numbers of CD8^+^ T-cells in the spleen at 7 and 14 days pi and in mesenteric lymph nodes from 7 to 50 days pi and by the elevation of CD4^+^ T cells, which peaked at 14 days pi in both compartments [34]. In contrast to that, in the present study, no conspicuous changes in the absolute numbers of the main lymphocyte subpopulations in various lymphatic organs during *E. cuniculi* infection in rabbits were detected. An increase in the absolute numbers of CD8^+^ lymphocytes was detected only in peripheral blood at 6 weeks pi. Furthermore, in Peyer’s patches, a significant increase in relative numbers of CD8^+^ lymphocytes 4 weeks pi and an increase in CD4^+^; CD8^+^; CD4^+^8^+^ and pT^+^ lymphocytes 6 weeks pi were detected.

In order to determine the commencement of antigen-specific cell response, proliferation of lymphocytes isolated from various organs after *E. cuniculi* stimulation in vitro was studied. A significant specific cell response was detected in peripheral blood at 4 weeks pi, in the spleen from 4 to 8 weeks pi, and in popliteal lymph nodes at 6 weeks pi. In contrast to that, in *E. cuniculi* infected mice, antigen-specific response of splenocytes was detected as early as 7 days pi and lasted up to the end of the experiment at 24 days pi. Moreover, proliferation ability after nonspecific stimulation with T-cell mitogens, Con A and PHA, was significantly lower in spleen cells from immunocompetent mice 7 days pi than in uninfected mouse spleen cells. At 14 days pi, the immune response to these T-cells mitogens returned to normal. The response to B-cell mitogens, LPS and PWM, remained unchanged at both times pi [35]. Khan and Moretto [36] also described transient immunosuppression, expressed as a decrease in Con A response at 17 and 24 days pi in C57BL/6 mice which are highly susceptible to encephalitozoonosis. Transient periods of lymphocyte hyporesponsiveness have also been observed for other parasitic infections such as *Neospora caninum* [37] and *Toxoplasma gondii* [38]. In rabbits, a decrease in mitogen-induced proliferation was detected only at 4 weeks pi in spleen cells after PWM stimulation and in Peyer’s patches after stimulation with PHA and PWM mitogens. When compared to mice, antigen-specific proliferation activity of *E. cuniculi* infected immunocompetent rabbit lymphocytes was delayed and transient suppression of non-specific proliferation activity was indistinct. Immunosuppressive effect of microsporidial infection is observed mainly during the early phase of infection, therefore 2 weeks pi were probably too late to detect lower proliferation ability. Rabbit encephalitozoonosis is an example of a balanced host-parasite relationship. Weak immune responses lead to clinical signs of disease or even to death as it occurs in immunodeficient mice. On the other hand, an excessive immune response may cause tissue damage, as appears to be the case in carnivores with encephalitozoonosis. Thus, it may be beneficial for the host to develop sufficient responses to allow microsporidial persistence, whereas immune reaction strong enough to eliminate *E. cuniculi* may cause more damage to the host than the mere presence of low numbers of the microsporidia [20, 35].

For detection of lymphocyte subpopulation responsible for antigen-specific proliferation a CFSE proliferation assay was performed in this study. In infected rabbits, CD4^+^ as well as CD8^+^ T cells proliferated significantly after stimulation with specific antigen. CD4^+^ T cells proliferation dominated at 2 weeks pi and CD8^+^ lymphocytes predominated at the end of the experimental period. Specific proliferation of IgM^+^ cells was detected only to a minor extent at the late stage of infection. Based on a murine model, the central role of cell-mediated immunity, especially that of CD8^+^ cytotoxic T lymphocytes, in defence against microsporidial infection, has been confirmed by adoptive transfer experiments [12, 18]. In mice after intraperitoneal infection, an early response of cytotoxic CD8^+^ T lymphocytes developed, reducing the parasite load by killing infected host cells via the perforin-dependent pathway [18]. Nevertheless, CD4^+^ T lymphocytes appear to play a very important role mainly after oral infection. Recently Moretto and Khan [39] reported that one of the roles of CD4^+^ T cells during microsporidial infection is their ability to secrete IL-21, which is needed for generation of robust effector CD8 T cell response. Salát et al. [40] proposed that CD8^+^-T lymphocyte-independent protection against the peroral route of infection is mediated by CD4^+^ T lymphocytes, producing IFN-γ and by B lymphocytes, producing specific antimicrosporidial antibody [33, 41]. IFN-γ is essential for the survival of mice infected either intraperitoneally or orally [17, 36] apparently because of its ability to polarize adaptive immunity toward a Th-1 type response, promoting the generation of CD8^+^ T-cell immunity. Recently, da Costa et al. [42] suggested the importance of B-1 lymphocytes in control of *E. cuniculi* infection. B-1 cells are preferentially located in the peritoneal and pleural cavities, are capable of IgM antibodies production and are antigen-presenting cells.

In the present study, a significant elevation of IFN-γ mRNA and polarization of the immune response towards Th1 were detected from 2 to 8 weeks pi in the spleen, mesenteric lymph nodes and Peyer’s patches of rabbits orally infected with *E. cuniculi.* This correlated with the recently described elevation of serum IFN-γ levels in naturally infected rabbits [19]. Similarly, *E. cuniculi* infection in immunocompetent mice induces a strong cellular immune response characterized by the production of IFN-γ. Mice unable to produce this cytokine are susceptible to infection [36]. There is evidence that IFN-γ, as proinflammatory cytokine, is a potent activator of macrophages, resulting in the effective killing of phagocytosed microsporidial spores by the production of toxic oxygen metabolites [43, 44]. Another mechanism for the antimicrosporidial activity of IFN-γ may be its ability to upregulate major histocompatibility complex class I molecules on antigen-presenting cells, thereby enhancing the quality of antigen presentation and generation of an antigen specific CD8^+^ CTL response and cytotoxic activity of natural killer cells. The protective effect of IFN-γ was confirmed in a therapy experiment [40]. Moretto et al. [45] described the predominant role of another Th1 cytokine IL-12 in the expansion of effector CD8^+^ T-cell immunity against *E. cuniculi* in mice infected intraperitoneally. On the other hand, according to Salát et al. [17], IFN-γ is an essential cytokine for induction of anti-microsporidial protective immunity irrespective of the route of infection. Whereas IL-12 can contribute to the polarization of the immune response towards Th1 cytokines, however, it is not essential for control of oral infection with *E. cuniculi* in mice.

In contrast to that, in mixed samples from the small intestine, the predominance of a Th2 cytokine response, as a consequence of a significant increase in expression of Th2 cytokine IL-4 and IL-10 and no elevation of IFN-γ mRNA were detected. Nevertheless, in intraepithelial lymphocytes (IELs) of orally infected mice, an increase in both IFN-γ and IL-10 was observed as early as 3 days pi and the levels of these cytokines were elevated until day 21 pi. IELs are a primary source of immune defence against oral *E. cuniculi* infection in mice. Due to their ability to produce IFN-γ and exhibit strong cytolytic property, these cells are able to impede parasite multiplication. To counterbalance the potential harmful effect of IFN-γ overproduction, IELs also play an immunoregulatory role, especially through the expression of anti-inflammatory cytokine IL-10 that suppresses cytokine production by the Th1 subset, including IFN-γ [34]. Recently, Nevárez-Garza et al. [46] also reported elevated expression of IL-10 mRNA in the brain tissue of naturally *E. cuniculi* infected rabbits. However, in serum of the same animals, levels of IL-10 were similar to the control uninfected rabbits [19]. In the present study, 2 weeks pi was probably too late to note an early Th1 response in the small intestine after oral infection of rabbits. Instead of that, the elevation of Th2 cytokine IL-4, which presumably induces humoral immunity, was detected at 4, 6 and 8 weeks pi. As described previously in mice, humoral immunity is part of complex defence mechanisms against microsporidiosis. The main barrier against multiplication of microsporidia after oral infection is probably IELs, and specific antibodies produced in the intestine are able to enhance its effect [47]. In *E. cuniculi* infected rabbits, production of specific antibodies has been well documented. The specific IgM antibodies in serum were detected as early as 1 week after infection, with the commencement of the production of IgG antibodies 1 week later [7].

Information related to the role of the Th17 immune response in microsporidial infection is insufficient. IL-17 and other Th17 cytokines are essential for host defence, particularly against extracellular bacteria and fungi. However, their importance in protection against many intracellular pathogens was revealed as well [48, 49]. As microsporidia belong to fungi [2], the production of IL-17 during encephalitozoonosis in rabbits was noteworthy. Despite the fact that mRNA for IL-17 was found at detectable levels in all of the experimental animals, significantly increased expression was found only in splenocytes at 4 and 6 weeks pi and in the small intestine 4, 6 and 8 weeks pi. Moreover, in the small intestine 8 weeks pi, Th17 immune response exceeded that of Th1. Nevertheless, the Th17 lineage seems to play only a minor role in the course of *E. cuniculi* infection in rabbits. In the murine model of encephalitozoonosis, IL-17 cytokine was either under the detectable levels [42, 50] or serum levels of IL-17 as well as other cytokines (TNF-α and IL-2) increased in *E. cuniculi* infected mice that received B-1 cells as an evidence of B-1 cells role in the increase of pro-inflammatory cytokines [51].

Based on the present results, we can conclude that oral *E. cuniculi* infection in immunocompetent rabbits caused subclinical infection with the activation of humoral and cell-mediated immune responses. Dissemination of microsporidia in the body of the rabbit, especially to nervous tissue, is more rapid than previously reported, as *E. cuniculi* was detected in various nervous tissue as early as 2 weeks pi. Cell-mediated immunity was characterized by ability of both CD4^+^ and CD8^+^ T cells to proliferate after stimulation with specific antigens. Th1 polarization of immune response with a predominance of IFN-γ expression was detected in spleen, mesenteric lymph nodes and Peyer’s patches. The increased expression of IL-4 and IL-10 mRNA in mixed samples from the small intestine is indicative of balanced control of IFN-γ, which prevents tissue damage. On the other hand, it can play role in ability of *E. cuniculi* to survive and persist in the host organism in a balanced host-parasite relationship. The Th17 immunity lineage seems to play only a minor role in rabbit encephalitozoonosis.

## Data Availability

The datasets supporting the conclusions of this article are included within the article.

## References

[1] Didier ES, Didier PJ, Snowden KF, Shadduck JA (2000). Microsporidiosis in mammals. Microb Infect.

[2] Katinka MD, Duprat S, Cornillot E, Méténier G, Thomarat F, Prensier G, Barbe V, Peyretaillade E, Brottier P, Wincker P, Delbac F, El Alaoui H, Peyret P, Saurin W, Gouy M, Weissenbach J, Vivarès CP (2001). Genome sequence and gene compaction of the eukaryote parasite *Encephalitozoon cuniculi*. Nature.

[3] Deplazes P, Mathis A, Baumgartner R, Tanner I, Weber R (1996). Immunologic and molecular characteristics of *Encephalitozoon*—like microsporidia isolated form humans and rabbits indicate that *Encephalitozoon cuniculi* is a zoonotic parasite. Clin Infect Dis.

[4] Mathis A, Weber R, Deplazes P (2005). Zoonotic potential of the microsporidia. Clin Microbiol Rev.

[5] Hunt RD, King NW, Foster HL (1972). Encephalitozoonosis: evidence for vertical transmission. J Infect Dis.

[6] Baneux PJ, Pognan F (2003). In utero transmission of *Encephalitozoon cuniculi* strain type I in rabbits. Lab Anim.

[7] Jeklova E, Leva L, Kovarcik K, Matiasovic J, Kummer V, Kummer V, Maskova J, Skoric M, Faldyna M (2010). Experimental oral and ocular *Encephalitozoon cuniculi* infection in rabbits. Parasitology.

[8] Ozkan O, Karagoz A, Kocak N (2019). First molecular evidence of ocular transmission of Encephalitozoonosis during the intrauterine period in rabbits. Parasitol Int.

[9] Jeklova E, Leva L, Kummer V, Jekl V, Faldyna M (2019). Immunohistochemical detection of *Encephalitozoon cuniculi* in ocular structures of immunocompetent rabbits. Animals (Basel).

[10] Didier ES, Stovall ME, Green LC, Brindley PJ, Sestak K, Didier PJ (2004). Epidemiology of microsporidiosis: sources and modes of transmission. Vet Parasitol.

[11] Cox JC, Hamilton RC, Attwood HD (1979). An investigation of the route and progression of *Encephalitozoon cuniculi* infection in adult rabbits. J Protozool.

[12] Schmidt EC, Shadduck JA (1983). Murine encephalitozoonosis model for studying the host-parasite relationship of the chronic infection. Infect Immun.

[13] Shadduck JA, Orenstein JM (1993). Comparative pathology of microsporidiosis. Arch Pathol Lab Med.

[14] Harcourt-Brown FM, Holloway HKR (2003). *Encephalitozoon cuniculi* in pet rabbits. Vet Rec.

[15] Künzel F, Joachim A (2010). Encephalitozoonosis in rabbits. Parasitol Res.

[16] Braunfuchsová P, Salát J, Kopecký J (2002). Comparison of the significance of CD4+ and CD8+ T lymphocytes in the protection of mice against *Encephalitozoon cuniculi* infection. J Parasitol.

[17] Salát J, Sak B, Le T, Kopecký J (2004). Susceptibility of IFN-gamma or IL-12 knock-out and SCID mice to infection with two microsporidian species, *Encephalitozoon cuniculi* and *E. intestinalis*. Folia Parasitol (Praha).

[18] Khan IA, Schwartzman JD, Kasper LH, Moretto M (1999). CD8 + CTLs are essential for protective immunity against *Encephalitozoon cuniculi* infection. J Immunol.

[19] Rodríguez-Tovar LE, Castillo-Velázquez U, Arce-Mendoza AY, Nevárez-Garza AM, Zarate-Ramos JJ, Hernández-Vidal G, Rodríguez-Ramírez HG, Trejo-Chávez A (2019). Interferon γ and interleukin 10 responses in immunocompetent and immunosuppressed New Zealand White rabbits naturally infected with *Encephalitozoon cuniculi*. Dev Comp Immunol.

[20] Schmidt EC, Shadduck JA (1984). Mechanisms of resistance to the intracellular protozoan *Encephalitozoon cuniculi* in mice. J Immunol.

[21] Sak B, Saková K, Ditrich O (2004). Effects of a novel anti-exospore monoclonal antibody on microsporidial development in vitro. Parasitol Res.

[22] Visvesvara GS, Moura H, Leitch J, Schwartz DA, Wittner M, Weiss LM (1999). Culture and propagation of microsporidia. The microsporidia and microsporidiosis.

[23] Mo L, Drancourt M (2004). Monoclonal antibodies for specific detection of *Encephalitozoon cuniculi*. Clin Diagn Lab Immunol.

[24] Jass A, Matiasek K, Hartmann K, Kuchenhoff H, Fischer A (2006). Evaluierung von Liquoruntersuchung und PCR zur Diagnose der Enzephalitozoonose beim Kaninchen. Prakt Tierartz.

[25] Jeklova E, Jekl V, Kovarcik K, Hauptman K, Koudela B, Neumayerova H, Knotek Z, Faldyna M (2010). Usefulness of detection of specific IgM and IgG antibodies for diagnosis of clinical encephalitozoonosis in pet rabbits. Vet Parasitol.

[26] Jeklova E, Leva L, Faldyna M (2007). Lymphoid organ development in rabbits: major lymphocyte subsets. Dev Comp Immunol.

[27] Vandesompele J, De Preter K, Pattyn F, Poppe B, Van Roy N, De Paepe A, Speleman F (2002). Accurate normalization of real-time quantitative RT-PCR data by geometric averaging of multiple internal control genes. Genome Biol.

[28] Didier ES, Weiss LM, Becnel JJ (2014). Mammalian animal models of human microsporidiosis. Microsporidia. Pathogens of Opportunity.

[29] Kunstýr I, Lev L, Naumannm S (1986). Humoral antibody response of rabbits to experimental infection with *Encephalitozoon cuniculi*. Vet Parasitol.

[30] Wicher V, Baughn RE, Fuentealba C, Shadduck JA, Abbruscato F, Wicher K (1991). Enteric infection with an obligate intracellular parasite, *Encephalitozoon cuniculi*, in an experimental model. Infect Immun.

[31] Csokai J, Joachim A, Gruber A, Tichy A, Pakozdy A, Künzel F (2009). Diagnostic markers for encephalitozoonosis in pet rabbits. Vet Parasitol.

[32] Künzel F, Gruber A, Tichy A, Edelhofer R, Nell B, Hassan J, Leschnik M, Thalhammer JG, Joachim A (2008). Clinical symptoms and diagnosis of encephalitozoonosis in pet rabbits. Vet Parasitol.

[33] Sak B, Salát J, Horká H, Saková K, Ditrich O (2006). Antibodies enhance the protective effect of CD4+ T lymphocytes in SCID mice perorally infected with *Encephalitozoon cuniculi*. Parasite Immunol.

[34] Moretto M, Weiss LM, Khan IA (2004). Induction of a rapid and strong antigen-specific intraepithelial lymphocyte response during oral *Encephalitozoon cuniculi* infection. J Immunol.

[35] Didier ES, Shadduck JA (1988). Modulated immune responsiveness associated with experimental *Encephalitozoon cuniculi* infection in BALB/c mice. Lab Anim Sci.

[36] Khan IA, Moretto M (1999). Role of gamma interferon in cellular immune response against murine *Encephalitozoon cuniculi* infection. Infect Immun.

[37] Khan IA, Schwartzman JD, Fonseka S, Kasper LH (1997). Neospora caninum: role for immune cytokines in host immunity. Exp Parasitol.

[38] Sternberg J, McGuigan F (1992). Nitric oxide mediates suppression of T cell responses in murine *Trypanosoma brucei* infection. Eur J Immunol.

[39] Moretto MM, Khan IA (2016). IL-21 is important for induction of KLRG1+ effector CD8 T cells during acute intracellular infection. J Immunol.

[40] Salát J, Jelínek J, Chmelař J, Kopecký J (2008). Efficiency of gamma interferon and specific antibody for treatment of microsporidiosis caused by *Encephalitozoon cuniculi* in SCID mice. Antimicrob Agents Chemother.

[41] Salát J, Horka H, Sak B, Kopecky J (2006). Pure CD4+ T lymphocytes fail to protect perorally infected SCID mice from lethal microsporidiosis caused by *Encephalitozoon cuniculi*. Parasite Res.

[42] da Costa LFV, Alvares-Saraiva AM, Dell’Armelina Rocha PR, Spadacci-Morena DD, Perez EC, Mariano M, Lallo MA (2017). B-1 cell decreases susceptibility to encephalitozoonosis in mice. Immunobiology.

[43] Didier ES, Shadduck JA (1994). IFN-gamma and LPS induce murine macrophages to kill *Encephalitozoon cuniculi* in vitro. J Eukaryot Microbiol.

[44] Didier ES (1995). Reactive nitrogen intermediates implicated in the inhibition of *Encephalitozoon cuniculi* (phylum Microspora) replication in murine peritoneal macrophages. Parasite Immunol.

[45] Moretto MM, Lawlor EM, Khan IA (2010). Lack of interleukin-12 in p40-deficient mice leads to poor CD8+ T-cell immunity against *Encephalitozoon cuniculi* infection. Infect Immun.

[46] Nevárez-Garza AM, Castillo-Velázquez U, Soto-Domínguez A, Montes-de-Oca-Luna R, Zamora-Ávila DE, Wong-González A, Rodríguez-Tovar LE (2018). Quantitative analysis of TNF-α, IL-4, and IL-10 expression, nitric oxide response, and apoptosis in *Encephalitozoon cuniculi*-infected rabbits. Dev Comp Immunol.

[47] Sak B, Ditrich O (2005). Humoral intestinal immunity against *Encephalitozoon cuniculi* (Microsporidia) infection in mice. Folia Parasitol (Praha).

[48] Curtis MM, Way SS (2009). Interleukin-17 in host defence against bacterial, mycobacterial and fungal pathogens. Immunology.

[49] Mensikova M, Stepanova H, Faldyna M (2013). Interleukin-17 in veterinary animal species and its role in various diseases: a review. Cytokine.

[50] Francisco Neto A, Dell’Armelina Rocha PR, Perez EC, Xavier JG, Peres GB, Spadacci-Morena DD, Alvares-Saraiva AM, Lallo MA (2017). Diabetes mellitus increases the susceptibility to encephalitozoonosis in mice. PLoS One.

[51] Langanke Dos Santos D, Alvares-Saraiva AM, Xavier JG, Spadacci-Morena DD, Peres GB, Dell’Armelina Rocha PR, Perez EC, Lallo MA (2018). B-1 cells upregulate CD8 T lymphocytes and increase proinflammatory cytokines serum levels in oral encephalitozoonosis. Microbes Infect.

